# Hepatic arterial phase and portal venous phase computed tomography for dose calculation of stereotactic body radiation therapy plans in liver cancer: a dosimetric comparison study

**DOI:** 10.1186/1748-717X-8-264

**Published:** 2013-11-09

**Authors:** Jianghong Xiao, Yan Li, Qingfeng Jiang, Lan Sun, Fraser Henderson Jr, Yongsheng Wang, Xiaoqin Jiang, Guangjun Li, Nianyong Chen

**Affiliations:** 1 Department of Radiation Oncology, Cancer Center, West China Hospital, Sichuan University, Chengdu, Sichuan 610041, PR China; 2 Center for Radiation Physics and Technology, Cancer Center, West China Hospital, Sichuan University, Chengdu, Sichuan 610041, PR China; 3 University of Virginia School of Medicine, Charlottesville, Va 22908, USA

**Keywords:** Liver cancer, Contrast agent, Dose calculation, Hepatic arterial phase CT, Portal venous phase CT, Stereotactic body radiation therapy, Volumetric modulated arc therapy

## Abstract

**Purpose:**

To investigate the effect of computed tomography (CT) using hepatic arterial phase (HAP) and portal venous phase (PVP) contrast on dose calculation of stereotactic body radiation therapy (SBRT) for liver cancer.

**Methods:**

Twenty-one patients with liver cancer were studied. HAP, PVP and non-enhanced CTs were performed on subjects scanned in identical positions under active breathing control (ABC). SBRT plans were generated using seven-field three-dimensional conformal radiotherapy (7 F-3D-CRT), seven-field intensity-modulated radiotherapy (7 F-IMRT) and single-arc volumetric modulated arc therapy (VMAT) based on the PVP CT. Plans were copied to the HAP and non-enhanced CTs. Radiation doses calculated from the three phases of CTs were compared with respect to the planning target volume (PTV) and the organs at risk (OAR) using the Friedman test and the Wilcoxon signed ranks test.

**Results:**

SBRT plans calculated from either PVP or HAP CT, including 3D-CRT, IMRT and VMAT plans, demonstrated significantly lower (*p* <0.05) minimum absorbed doses covering 98%, 95%, 50% and 2% of PTV (D98%, D95%, D50% and D2%) than those calculated from non-enhanced CT. The mean differences between PVP or HAP CT and non-enhanced CT were less than 2% and 1% respectively. All mean dose differences between the three phases of CTs for OARs were less than 2%.

**Conclusions:**

Our data indicate that though the differences in dose calculation between contrast phases are not clinically relevant, dose underestimation (IE, delivery of higher-than-intended doses) resulting from CT using PVP contrast is larger than that resulting from CT using HAP contrast when compared against doses based upon non-contrast CT in SBRT treatment of liver cancer using VMAT, IMRT or 3D-CRT.

## Introduction

Hepatocellular carcinoma (HCC) is the fifth most common cancer worldwide [1]. Moreover, liver is a common site for metastases from a variety of primary malignancies [2]. Generally, surgery is acknowledged to be the most effective treatment for liver cancer, though only 20-40% of HCC patients may benefit from radical therapies [3]. By time of diagnosis only 10-20% of metastatic liver cancer cases undergo surgical resection [4]. However, stereotactic body radiation therapy (SBRT), which includes the application of volumetric modulated arc therapy (VMAT), has been an important addition to the arsenal for treatment of liver cancer [5-7].

SBRT is a technique designed to deliver radiation precisely to delineated tumor areas. However, tactics used to define a gross tumor volume (GTV) might vary significantly among professionals, centers and levels of experiences [8]. Use of contrast in hepatic arterial phase (HAP) and portal venous phase (PVP) computed tomography (CT) has improved detection accuracy in liver cancer [9,10]. However, contrasted imaging may adversely influence the prescribed SBRT dose distribution. To quantify the influence of contrast on dose calculation, two modes of investigation have been used. One is based on phantoms, or mathematical algorithms, designed to take into account parameters that include photon beam energies, molarities and contrast agent expansion [11,12]. Human studies are another mode, and they have revealed a negligible effect on dose calculation in regions with low contrast agent penetration [13-15]. However, it was reported that in treatment of upper abdominal tumors contrast agent is responsible for greater than 2% increase in monitor units (MUs) of three-dimensional conformal radiotherapy (3D-CRT) [16], but that the effect of contrast agent on dose calculation decreased with an increasing number of incident beams [12]. Since VMAT plans are designed using a larger number of segments (typically, there are 91 segments in a single-arc VMAT plan), the effect of contrast agent on VMAT plans is unknown.

Because liver has a dual blood supply from the hepatic artery and portal vein, distribution of contrast agent should differ between HAP and PVP CTs. It would then be reasonable to suspect that radiotherapy plans, based on either HAP or PVP CT, may differ as well and that dosing errors may result. To our knowledge, there are no studies on the effect of different CT phases for dose calculation of SBRT treatment plans, especially using VMAT, in liver cancer. We therefore conducted this study to query the influence of HAP and PVP CT on dose calculation of SBRT plans in liver cancer.

## Material and methods

### Eligibility

Twenty-one liver cancer patients were enrolled from a single institution. Included patients had no more than three lesions, and liver lesions were no larger than 6 cm in diameter. All patients were ruled-out for surgical resection before being enrolled (Table 1).

**Table 1:** Basic and clinical characteristics of selected patients in present study (n = 21)

**Age**	
Median	48
Range	32-77
Gender	
Male	15 (71.4%)
Female	6 (29.6%)
Pathology	
Primary hepatocellular carcinoma	9 (42.9%)
Metastatic liver cancer	12 (57.1%)
PTV Volume (cm^3^)	
0-- < 50	14 (66.7%)
50-- < 100	4 (19.0%)
100-- < 150	2 (9.5%)
150--200	1 (4.8%)

### Acquisition of CT

Each patient was immobilized in a stereotactic body frame (SBF, Elekta Oncology System, Sweden) in the supine position with arms raised above the head. Patients were trained to adapt to active breathing control (ABC) beforehand at moderate deep inspiration breath-hold with 75% of maximum inspiratory volume. Non-enhanced CT covering the total liver volume was obtained (Philips Gemini GXL, 120 kVp, 90 mAs) at 3 mm slice thickness under ABC. 25–30 seconds after intravenous bolus injection of 120 ml of the contrast agent (Iopamiro370, a non-ionic X-ray contrast agent; main ingredient: iopamidol 370 mgI/ml, Bracco S.P.A., Italy) with a power injector at a rate of 4–5 ml/second, the HAP CT was scanned; PVP CT was obtained 55–60 seconds after the injection.

### Region of interest contouring

**Figure 1:**
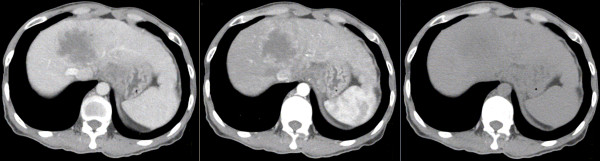
**Three phases of CTs for the same patient.** Left: portal venous phase (PVP) CT; Middle: hepatic arterial phase (HAP) CT; Right: non-enhanced CT.

After the image acquisition, CTs obtained in three phases were transferred to a radiotherapy planning system (Pinnacle3 9.0, Philips Inc., USA). Representative imaging demonstrating the three phases is provided in Figure 1.

The GTV and organs at risk (OAR) were delineated on the PVP CT. GTV was expanded by a 5-mm radial and a 10-mm cranial-caudal margin to create the planning target volume (PTV). Liver, spinal cord, stomach, bowel and kidneys were contoured as the OARs.

### Treatment planning

SBRT plans were designed on the PVP CT using 3D-CRT, IMRT and VMAT respectively for each patient. All plans were executed using a 6 MV photon beam from a linear accelerator (Synergy, Elekta, Sweden). The prescription doses were 48 Gy in 4 fractions.

3D-CRT plans were generated using seven coplanar static-fields. According to the guidance from RTOG 0236 recommendations for lung SBRT, no additional margin at the edges of the blocks or multi-leaf collimator (MLC) jaws beyond the PTV was considered. In this study, the minimum absorbed dose covering 95% of PTV (D95%) met or exceeded 48 Gy; total patient tissue volume (not including PTV) receiving ≥110% of the prescription dose could not exceed 1.0 cm^3^; liver mean dose (LMD) was less than 22 Gy; the percentage of right kidney volume receiving ≥15 Gy (V15) was less than 33%; dose to spinal cord could not exceed 18 Gy; maximum volume of bowel and stomach receiving ≥30 Gy (V30) less than 0.5 cm^3^.

IMRT plans (7 F-IMRT) were generated using the same fields as the 3D-CRT plans, optimized using Direct Machine Parameter Optimization (DMPO). Additional parameters were as follows: the minimum segment area was 4 cm^2^; the minimum segment MU was 5; the maximum number of segments was 50; total patient tissue volume receiving ≥110% of the prescription dose could not exceed 1.0 cm^3^. Remaining parameters were the same as in 3D-CRT plans.

VMAT plans were generated using a single arc (181°-180°). Other parameters were identical to those of the IMRT plan.

All doses were calculated using a collapsed-cone convolution (CC) algorithm with grid size 0.4 × 0.4 × 0.4 cm^3^, and all doses were calculated using the electron densities converted from CT number (HU) based on the Model 062 M Electron density phantom composed of eight different tissue equivalent inserts (doses to tissue). After the completion of plans using PVP CT, prescription doses were normalized to MUs. All contours and plans were copied to the HAP and non-enhanced CTs. Thereafter, the radiation doses were recalculated.

To verify dose differences caused by contrast agent or other factors (such as algorithm, inconsistency of patients’ positions, etc.), the density of three phases of CTs (PVP, HAP and non-enhanced) were specified as the density of water. Then the doses were recalculated (doses to water).

### Statistical analysis

To evaluate the equivalence of the patients’ positions and coordinates among these CTs after image fusion, Y coordinates (cranial-caudal axis) of the diaphragmatic dome and the source to surface distances (SSD) were recorded and analyzed. Dose parameters in the targets and OARs among these CTs were compared, including the D98%, D95%, D50%, D2%, heterogeneity index (HI = (D2% - D98%)/D50%) and conformity index (CI) where CI is given by the following equation: CI = (TV_95%_ × TV_95%_)/(TV × V_95%_)) [N.B. TV_95%_ = PTV volume covered by the 95% isodose, TV = PTV, and V_95%_ = volume of the 95% isodose]. OAR parameters included the D2% of the right kidney, bowel, stomach and spinal cord; the mean dose of the liver and right kidney; and the percentage of total liver volume receiving ≥21 Gy (V21). GTV was excluded from liver volume. Differences were analyzed by Friedman test or Wilcoxon signed ranks test (SPSS, Release 17.0). *p* < 0.05 (2-tailed) indicated statistical significance.

## Results

### SSD

Differences between SSDs ranged from -8.7 to 8.50 mm in each beam. Average SSD difference between PVP CT and non-enhanced CT was 0.23 ± 1.77 mm and for HAP CT versus non-enhanced CT was 0.28 ± 1.77 mm. Diaphragmatic dome coordinates differed with a range of -9.0 to 6.0 mm. Average difference of the diaphragmatic dome coordinate was 0.86 ± 4.15 mm for PVP CT vs. non-enhanced CT, and -0.14 ± 4.60 mm for HAP CT vs. non-enhanced CT. There were no significant differences among the SSDs or diaphragmatic dome coordinate of the PVP, HAP and non-enhanced CTs (*p* > 0.05).

Hounsfield units (HU) representing the GTV and liver varied significantly (*p* < 0.05) among the PVP, HAP, and non-enhanced CTs. HU values for GTV and liver by imaging phase used, in terms of magnitude, were PVP CT > HAP CT > non-enhanced CT. The mean differences of HUs in GTV and liver among three sets of CTs are as follows: 41.06 ± 24.74 HU and 58.05 ± 17.83 HU for PVP CT vs. non-enhanced CT, 22.58 ± 17.62 HU and 20.23 ± 22.56 HU for the set of HAP CT vs. non-enhanced CT, and 18.48 ± 14.65 HU and 37.82 ± 14.82 HU for the phase of PVP CT vs. HAP CT.

### PTV

We calculated significant variation (*p* < 0.05) in D98%, D95%, D50% and D2% among PVP-, HAP- and non-enhanced CT-based plans, regardless of whether 3D-CRT, IMRT or VMAT was used. D98%, D95%, D50% and D2% calculated from the three phases of CTs, by order of magnitude, were as follows: PVP CT < HAP CT < non-enhanced CT (*p* < 0.05). Maximum differences in D98%, D95%, D50% and D2% calculated from the plans with three phases of CTs were, respectively, 2.75%, 2.66%, 2.94%, 4.00% (PVP CT vs. non-enhanced CT); 2.10%, 2.15%, 2.43%, 2.72% (HAP CT vs. non-enhanced CT); and 1.93%, 1.86%, 1.90%, 2.99% (PVP CT vs. HAP CT). Mean differences (MD to tissue), however, were less at 2% (PVP CT vs. non-enhanced CT), 1% (HAP CT vs. non-enhanced CT), and 1% (PVP CT vs. HAP CT) (see Table 2).

**Table 2:** Comparison of PTV doses calculated from PVP, HAP and non-enhanced CTs

	**Dp**	**Da**	**Dn**	P(p,a,n)*	**(Dn-Dp)/Dn(%)**	**P(p,n)#**	**(Dn-Da)/Dn(%)**	**P(a,n)#**	**(Da-Dp)/Dn(%)**	**P(p,a)#**
**3D-CRT**										
CI	0.84 ± 0.07	0.83 ± 0.07	0.83 ± 0.07	0.116	-1.15 ± 2.44	-	-0.49 ± 1.72	-	-0.66 ± 1.00	-
HI	0.17 ± 0.02	0.17 ± 0.02	0.17 ± 0.02	0.117	1.04 ± 4.84	-	-0.50 ± 5.77	-	1.54 ± 4.29	-
D98% (Gy)	46.83 ± 0.28	47.09 ± 0.41	47.36 ± 0.46	0.000	1.12 ± 0.78	0.000	0.56 ± 0.51	0.000	0.56 ± 0.58	0.000
D95% (Gy)	48.09 ± 0.06	48.36 ± 0.29	48.62 ± 0.40	0.000	1.10 ± 0.76	0.000	0.54 ± 0.58	0.000	0.56 ± 0.56	0.000
D50% (Gy)	53.17 ± 0.52	53.52 ± 0.57	53.83 ± 0.75	0.000	1.23 ± 0.81	0.000	0.58 ± 0.54	0.000	0.64 ± 0.56	0.000
D2% (Gy)	55.63 ± 1.14	56.09 ± 1.28	56.39 ± 1.43	0.000	1.33 ± 1.21	0.000	0.53 ± 1.09	0.000	0.81 ± 0.93	0.000
**IMRT**										
CI	0.85 ± 0.04	0.82 ± 0.06	0.79 ± 0.09	0.000	-8.60 ± 9.78	0.000	-4.34 ± 6.09	0.000	-4.25 ± 5.84	0.000
HI	0.05 ± 0.01	0.06 ± 0.01	0.06 ± 0.01	0.040	4.82 ± 8.30	0.006	2.58 ± 5.74	0.070	2.24 ± 5.70	0.111
D98% (Gy)	47.60 ± 0.20	47.85 ± 0.30	48.06 ± 0.45	0.000	0.94 ± 0.68	0.000	0.43 ± 0.49	0.000	0.51 ± 0.43	0.000
D95% (Gy)	48.05 ± 0.08	48.31 ± 0.23	48.54 ± 0.35	0.000	1.01 ± 0.72	0.000	0.46 ± 0.50	0.000	0.55 ± 0.51	0.000
D50% (Gy)	49.24 ± 0.30	49.54 ± 0.43	49.80 ± 0.48	0.000	1.11 ± 0.83	0.000	0.52 ± 0.62	0.000	0.59 ± 0.53	0.000
D2% (Gy)	50.28 ± 0.45	50.61 ± 0.51	50.91 ± 0.60	0.000	1.23 ± 0.99	0.000	0.59 ± 0.74	0.000	0.64 ± 0.66	0.000
**VMAT**										
CI	0.81 ± 0.08	0.79 ± 0.08	0.78 ± 0.10	0.000	-4.93 ± 6.74	0.000	-2.38 ± 4.89	0.002	-2.54 ± 2.91	0.000
HI	0.07 ± 0.02	0.08 ± 0.02	0.08 ± 0.02	0.137	1.92 ± 6.65	-	-0.51 ± 4.73	-	2.43 ± 4.11	-
D98% (Gy)	47.45 ± 0.23	47.67 ± 0.32	47.89 ± 0.47	0.000	0.90 ± 0.64	0.000	0.45 ± 0.45	0.000	0.45 ± 0.38	0.000
D95% (Gy)	48.06 ± 0.04	48.28 ± 0.21	48.51 ± 0.31	0.000	0.93 ± 0.66	0.000	0.47 ± 0.42	0.000	0.46 ± 0.44	0.000
D50% (Gy)	49.98 ± 0.41	50.27 ± 0.51	50.52 ± 0.51	0.000	1.06 ± 0.79	0.000	0.49 ± 0.51	0.000	0.58 ± 0.52	0.000
D2% (Gy)	51.19 ± 0.62	51.54 ± 0.76	51.78 ± 0.80	0.000	1.13 ± 0.87	0.000	0.46 ± 0.53	0.000	0.68 ± 0.59	0.000

***Abbrevations***: PVP, portal venous phase; HAP, hepatic arterial phase. Dp, Da and Dn were the dose calculated from portal venous phase, hepatic arterial phase and non-enhanced phase CTs respectively. 98%, 95%, 50% and 2%, were the minimum absorbed dose that covers 98%, 95%, 50% and 2% of the volume of the PTV respectively. CI, the conformity index; HI, the heterogeneity index. *, Friedman test; #, Wilcoxon signed ranks test.

### OARs

Analysis of doses to OAR shows LMD calculated from either PVP or HAP CT was significantly lower than those from the non-enhanced CT (*p* < 0.05). However, all mean differences across the three phases of CT in dose to OARs was less than 2% (Table 3).

**Table 3:** Comparison of OARs’ doses calculated from PVP, HAP and non-enhanced CTs

	**Dp**	**Da**	**Dn**	P(p,a,n)*	**(Dn-Dp)/Dn (%)**	**P(p,n)#**	**(Dn-Da)/Dn (%)**	**P(a,n)#**	**(Da-Dp)/Dn (%)**	**P(p,a)#**
**3D-CRT**										
Liver V21 (%)	12.77 ± 11.45	12.87 ± 11.53	12.94 ± 11.55	0.000	1.70 ± 1.50	0.000	0.90 ± 1.20	0.000	0.80 ± 0.85	0.000
Liver Dmean (Gy)	8.52 ± 5.00	8.57 ± 5.03	8.61 ± 5.04	0.000	1.02 ± 0.73	0.000	0.51 ± 0.64	0.000	0.52 ± 0.47	0.000
Right kidney D2%(Gy)	13.59 ± 14.36	13.60 ± 14.35	13.69 ± 14.48	0.000	0.56 ± 1.29	0.000	0.33 ± 1.10	0.001	0.23 ± 0.58	0.140
Right kidney Dmean (Gy)	2.87 ± 3.47	2.87 ± 3.47	2.89 ± 3.50	0.201	-0.09 ± 1.01	-	0.18 ± 0.81	-	-0.27 ± 0.53	-
Bowl D2% (Gy)	12.83 ± 8.63	12.84 ± 8.65	12.86 ± 8.72	0.040	-0.31 ± 2.50	0.254	-0.13 ± 2.27	0.074	-0.18 ± 1.13	0.268
Stomach D2% (Gy)	16.42 ± 7.62	16.54 ± 7.66	16.65 ± 7.69	0.000	1.45 ± 2.33	0.003	0.71 ± 1.40	0.004	0.74 ± 1.31	0.001
Spinal cord D2% (Gy)	11.28 ± 6.79	11.31 ± 6.79	11.36 ± 6.81	0.006	0.73 ± 1.38	0.000	0.46 ± 0.88	0.004	0.27 ± 1.06	0.234
**IMRT**										
Liver V21 (%)	25.31 ± 6.90	25.35 ± 6.89	25.39 ± 6.87	0.040	0.38 ± 0.87	0.046	0.19 ± 0.68	0.338	0.19 ± 0.43	0.026
Liver Dmean (Gy)	10.00 ± 4.95	10.05 ± 4.99	10.09 ± 4.99	0.000	0.97 ± 0.77	0.000	0.49 ± 0.74	0.000	0.48 ± 0.46	0.000
Right kidney D2% (Gy)	14.71 ± 14.88	14.71 ± 14.89	14.82 ± 15.02	0.003	0.32 ± 1.22	0.002	0.30 ± 1.21	0.006	0.01 ± 0.50	0.050
Right kidney Dmean (Gy)	3.04 ± 3.12	3.03 ± 3.12	3.06 ± 3.15	0.090	0.02 ± 1.00	-	0.29 ± 0.84	-	-0.27 ± 0.54	-
Bowl D2% (Gy)	10.99 ± 6.88	11.02 ± 6.90	11.09 ± 6.93	0.000	0.90 ± 0.86	0.000	0.65 ± 0.88	0.002	0.26 ± 0.69	0.178
Stomach D2% (Gy)	11.73 ± 6.99	11.80 ± 7.03	11.85 ± 7.07	0.000	0.89 ± 1.90	0.012	0.39 ± 1.23	0.008	0.50 ± 1.28	0.002
Spinal cord D2% (Gy)	9.09 ± 4.50	9.12 ± 4.49	9.17 ± 4.50	0.003	0.99 ± 1.64	0.003	0.57 ± 1.13	0.001	0.42 ± 0.95	0.195
**VMAT**										
Liver V21 (%)	20.71 ± 7.27	20.75 ± 7.27	20.77 ± 7.26	0.017	0.35 ± 0.71	0.018	0.14 ± 0.47	0.355	0.21 ± 0.34	0.005
Liver Dmean (Gy)	10.10 ± 5.24	10.16 ± 5.28	10.20 ± 5.29	0.000	0.97 ± 0.67	0.000	0.45 ± 0.59	0.000	0.52 ± 0.43	0.000
Right kidney D2% (Gy)	14.30 ± 14.80	14.33 ± 14.83	14.41 ± 14.87	0.000	0.68 ± 1.08	0.000	0.38 ± 0.89	0.000	0.31 ± 0.48	0.000
Right kidney Dmean (Gy)	3.34 ± 3.55	3.34 ± 3.55	3.36 ± 3.56	0.187	-0.03 ± 1.03	-	0.14 ± 0.76	-	-0.17 ± 0.41	-
Bowl D2% (Gy)	11.87 ± 7.28	11.90 ± 7.32	11.98 ± 7.35	0.000	0.95 ± 0.87	0.000	0.81 ± 1.02	0.001	0.14 ± 0.94	0.206
Stomach D2% (Gy)	11.53 ± 8.30	11.65 ± 8.47	11.65 ± 8.40	0.000	0.78 ± 1.60	0.001	0.08 ± 1.95	0.031	0.70 ± 1.12	0.000
Spinal cord D2% (Gy)	9.92 ± 3.79	9.93 ± 3.79	9.95 ± 3.80	0.013	0.24 ± 1.18	0.010	0.21 ± 0.56	0.031	0.04 ± 0.77	0.081

***Abbrevations***: V21, the percentage liver volume received more than 21 Gy; Dmean, the mean dose; D2%, the minimum absorbed dose that covers 2% of the volume of a certain OAR. The others as in Table 2. *, Friedman test; #, Wilcoxon signed ranks test.

With the density specified as the density of water, the mean differences in D98%, D95%, D50% and D2% for PTV (MD to water) among the three phases of CTs were less than 0.1%; the ratio of MD to water and MD to tissue were less than 6.64%.

Dose differences attributable to the contrast agent were similar among the three kinds of plans (single arc VMAT, 7 F-IMRT and 7 F-3D-CRT, *p* > 0.05) according to the Friedman test.

## Discussion

The liver possesses a dual blood supply from the hepatic portal vein and hepatic arteries. The hepatic portal vein supplies approximately 75% of the liver’s blood flow, and the hepatic artery accounts for the remainder. Moreover, the liver is the dominant organ for congregation of contrast agent. After intravenous bolus injection, contrast agent transported via hepatic artery arrives 20–30 seconds earlier to the liver than if transported via portal vein, and this differential explains the difference seen between HAP and PVP imaging. Studies have confirmed that utilizing both PVP and HAP CTs elevates the rates of detection [9,10]. Therefore, the PVP and HAP CTs are often applied during the simulation stage of liver cancer radiotherapy treatment. Hounsfield units represent a tissue’s electron density via x-ray attenuation, and as such, are a critical and contributory component of dose calculation in the planning system. In this study, we found that the HUs of the GTV and liver in both PVP and HAP CTs were higher than those in the non-enhanced CT; moreover, the increase of HUs in the PVP CT was more obvious than that in other phases.

In this study, SSDs varied in patients similarly to our previous report on lung cancer [13], while there was more variation than what has been seen on head and neck cancer [14]. Due to the significant cranial-caudal displacement of liver resulting from respiratory motion [17], we assessed the Y-axes coordinates of the diaphragmatic dome. Both the mean value and range of the diaphragmatic dome’s displacement were much smaller with ABC than without it. There were no significant differences in either SSDs or displacement of the diaphragmatic dome among these phases of CTs. Therefore, patients were considered to have been scanned in nearly identical positions; the influence of positioning errors among these CTs was negligible.

Shibamoto *et al.* reported an increase of 51 ± 17 HU of enhanced liver CT over non-enhanced. Mean increases of MUs were more than 2%, and the maximum increase was 7.6 MU for upper abdominal tumors [16]. In contrast to their results, our data showed that in the SBRT plans, the dose differences to PTV between the PVP and non-enhanced CTs were lower than 2%; however, the dose differences to PTV between the HAP and non-enhanced CTs were lower than 1%. We speculate that ABC technique explains the divergence. ABC works to coordinate scanning with cycles in respiratory movement, and thus we feel that our analysis of the treatment effect of contrast agent in imaging is better assessed employing the ABC technology. Choi *et al.* found the GTV HUs of head and neck increased by an average of 55 HUs, and that PTV doses differed less than 1% (enhanced CT vs. non-enhanced CT) [14]. These dose differences are similar to the dose changes between the HAP and non-enhanced CTs; however, they are less than the dose differences between the PVP and non-enhanced CTs in our study. Here we speculate that this change in dose underestimation may be caused by contrast agent distribution varied in different phases. Moreover HU values by order of magnitude for GTV and liver were PVP CT > HAP CT > non-enhanced CT (*p* < 0.05). Therefore, the resulting data encourages us to further clarify the effect of contrast agent application on the dose underestimation for the plans of radiotherapy.

Ramm *et al.* had found in a phantom-based study that the effect of contrast agent decreased with an increase of the number of incident beams [12]. In Ramm *et al*’s study, the plans were created with a single photon beam or two opposing photon beams or an isocentric four-field box technique. The number of incident beams was less than that in our study (VMAT 91 segments; IMRT ≤50 segments; 3D-CRT 7 beams). Although the VMAT plans had the most segments or beams, compared with non-enhanced CT, the dose underestimations attributable to the contrast agent showed no significant difference across the three plan types (*p* > 0.05). It is possible that when the number of beams or segments increases to a certain threshold value, the dose effects caused by the contrast agent will tend to be consistent. This question deserves further investigation.

There was concern that the accuracy of the CC algorithm might affect the accuracy of dose underestimation in our study. Therefore calibrated ionization chamber and radiochromic EBT type films in a homogenous polystyrene phantom and in heterogeneous lung phantoms were used for evaluating the accuracy of the algorithm [18]. The result indicated some small difference between the CC and Monte Carlo (MC) algorithms in the measurements. Knöös *et al.* also reported that when the average dose in the PTV for prostate cases calculated by the MC was set to 100%, the average dose, D95%, and D5% of PTV calculated by the Pinnacle CC algorithm were 100%, 98.2%, and 101.4% respectively [19]. Because prostatic tissue and liver are similarly homogenous we believe that the algorithm is translatable from prostate to liver and that the effect of the algorithm on the dose calculation for SBRT plans using VMAT or IMRT or 3D CRT is negligible. The results in this study indicated that when the density of PVP, HAP, and non-enhanced CTs were specified as the density of water, the MD-to-water among three phases of CTs were less than 0.1%, the ratio of MD-to-water and MD-to-tissue were less than 6.64%. This finding further confirmed that the dose differences were due to the effect of using contrast agent and not the algorithm.

In conclusion, SBRT plans for treating liver cancer, whether with VMAT or IMRT or 3D-CRT, are susceptible to dose underestimations caused by contrast agent from the PVP CT that are larger than those from the HAP CT when compared with non-enhanced CT. Nevertheless, the detection rate in HAP CT is higher than that in PVP CT [9]. Therefore, it is still recommended HAP CT to be applied for patient simulation in the treatment planning in liver cancer.

## Competing interest

The authors declare that the article content was composed in the absence of any commercial or financial relationships that could be construed as a potential conflict of interest.

## Authors’ contributions

JX, YL, QJ and LS contributed equally to this work, participated in the design of the study, carried out the study, performed the statistical analysis, and drafted the manuscript; YW, XJ and GL: helped to carried out the study; FH: reviewed and edited the manuscript; NC: conceived, designed, and coordinated the study, and edited and reviewed the manuscript. All authors read and approved the final manuscript.
